# Mini-Review: Assessing the Potential Impact of Microneedle Technologies on Home Healthcare Applications

**DOI:** 10.3390/medicines5020050

**Published:** 2018-06-08

**Authors:** Aaron McConville, Catherine Hegarty, James Davis

**Affiliations:** School of Engineering, Ulster University, Jordanstown BT37 0QB, UK; mcconville-a4@ulster.ac.uk (A.M.); Hegarty-c19@ulster.ac.uk (C.H.)

**Keywords:** microneedle, transdermal, needlestick, sharps disposal, bloodborne pathogen

## Abstract

The increasing devolution of healthcare towards community care has meant that the management of many conditions is conducted within the home either by community nurses or by the patients themselves. The administration of medicines within home healthcare scenarios can however be problematic—especially when considering the delivery of medicines through injection. The possibility of needlestick injury (NSI) has become an ever-present hazard within healthcare settings, with a significant proportion of percutaneous injuries occurring during the handling and disposal of the needle. The emergence of transdermal microneedle systems, however, offers a potentially revolutionary advance and could dramatically improve safety—particularly within home healthcare where there are mounting concerns over the use and disposal of sharps. A mini-review of the advantages proffered by microneedle drug delivery technologies is presented and the potential impact on delivery of medicines within the home is critically appraised.

## 1. Introduction

**Figure 1:**
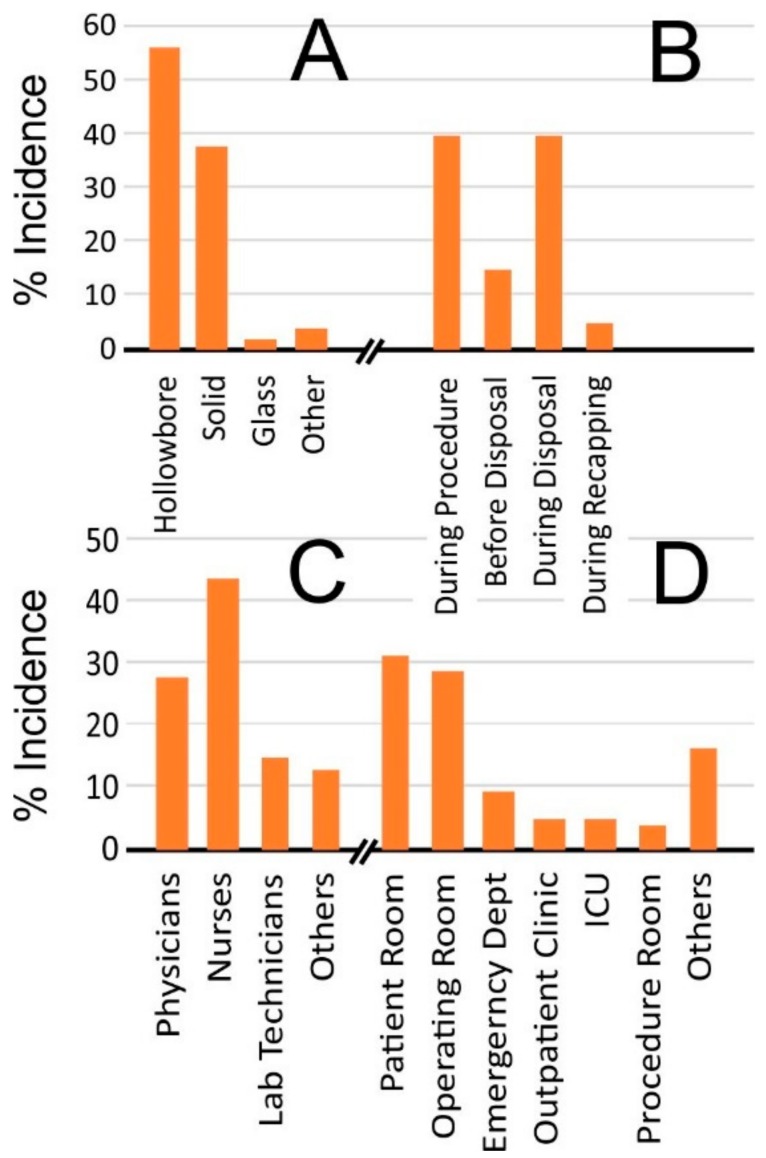
Incidence of needlestick injuries by device type (**A**), process (**B**), person (**C**), and location (**D**). (Figures adapted from Reference [4]).

Hypodermic injection is an invaluable clinical tool allowing the delivery of therapeutic agents that are otherwise unsuitable for administration through oral routes. However, the waste arising from the application of needles has long been a critical concern. There have been extensive surveys cataloguing the experiences of healthcare workers and the hazards posed by the use and disposal of needles [1,2,3]. In particular, the possibility of needlestick injury (NSI) has become an ever-present hazard within healthcare settings and is estimated to account for 80% of percutaneous injuries [4]. It is little surprise therefore to find that most countries have imposed a variety of legislative requirements to promote the adoption of safe working practices. The US Needlestick Safety and Prevention Act (2000) and the Occupational Safety and Health Administration (OSHA) Bloodborne Pathogen (BBP) Standard (2001) are notable in that it has been estimated that the incidence of sharps injuries dropped by some 34% shortly after their introduction [5,6]. Despite such advances, the eradication of NSI continues to be elusive and there remains considerable concern with an estimated 300,000 needlestick injuries reported annually in the US alone [7,8]. It must be noted that the true value is liable to be substantially higher, as it is widely acknowledged that many injuries go unreported [9]. A breakdown of NSI incidence by the Centres for Disease Control and Prevention [4], summarised in Figure 1, highlights that NSI events tend to fall within three main categories: during the procedure (40%), after use but before disposal (15%), and during disposal (40%).

A crucial aim of the regulatory instruments was to enhance safety through stipulating the mandatory provision of safety devices and sharps containers but, from the summary presented in Figure 1, it is clear that there is still some way to go in order to improve their efficacy. Advances in microneedle devices, typically in the form of patches, have garnered considerable interest in recent years as a potential alternative to hypodermic injections and are widely considered to possess a range of advantageous properties in relation to their clinical application [10,11]. While the needles are small, their ability to puncture the skin nevertheless renders them a potential sharps risk and their handling and disposal requires more than a modicum of caution. The latter is evidenced by a recent health notice from Public Health England (2017) highlighting concern over the use of microneedle rollers used in cosmetic practice [12]. Although it has been estimated that there are some 60 bloodborne pathogens, the majority of occupation-related infections are attributed to hepatitis B virus (HBV), hepatitis C virus (HCV), and human immunodeficiency virus (HIV) [13,14]. Upon penetrating the skin, the microneedles come in contact with the subject’s tissue and microcirculation, with the subsequent removal of the patch risking the retrieval of blood and/or serous fluid. Given the potential translation of microneedle systems to HHC contexts, it is vital therefore that the risk of accidental application and transmission of a BBP through careless use or disposal be acknowledged. The aim of this mini-review is to examine the present state of play in terms of sharps waste within both clinical and home healthcare environments and to provide a critical appraisal of the potential opportunities that microneedle devices proffer for reducing NSI events. There has been increasing interest in the latter in recent years and the technology is now at the point where there have been considerable advances in the design that could have far reaching consequences in terms of handling and waste management.

## 2. Home Healthcare

Needlestick injuries are not confined to clinical settings; it is within community and home healthcare that the hazards are becoming more apparent [15,16]. The increasing devolution and decentralisation of healthcare towards community care has meant that the management of many conditions is conducted within the home either by community nurses or by the patients themselves. Recent estimates suggest that there are some 3 million home healthcare workers in the US alone [17,18,19,20] and, with an annual impact of some $70 billion [19,20], is widely recognised as one of the fastest growing industries. The majority of HHC workers are classified as personal care assistants or home aides and, while their job specification may detail non-medical tasks [17,18], numerous surveys have highlighted the expansion of their role to assist with lancets or needles [21,22]. It is widely recognised that people are living longer with chronic conditions such as HIV and hepatitis and are receiving care in the home more frequently. As such, the potential for transmission of a BBP through accidental NSI is greatly increased [23]. The increasing concern is highlighted by reports that 35% of nurses and 6% of aides suffered at least one injury with a previously used sharp during their HHC career and that SEDs were uncommon [16].

**Figure 2:**
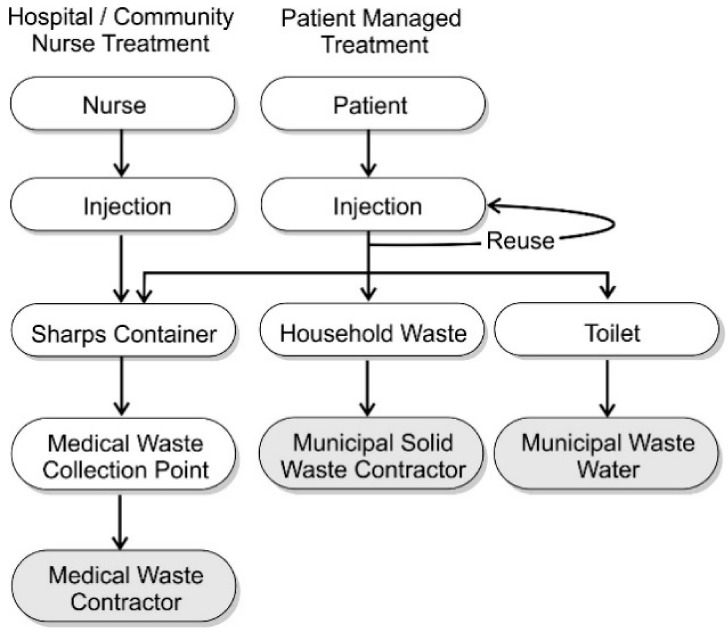
Sharps disposal practices for healthcare worker- and patient-managed treatment regimes.

The improvements that have been achieved thus far in reducing NSI incidence have come through professional responsibility and institutional accountability and, while OSHA standards still apply to treatment supplied by community care nurses, such frameworks are largely absent when considering patient-led treatment. It is inevitable that many will involve the use of parenteral injections (intravenous, subcutaneous, or intramuscular) and create a degree of concern over the methods of waste disposal and the accompanying sharps hazard. In the US, there are some 9 million patients receiving some form of healthcare at home that requires self-injection, and an estimated 3 billion used sharps are regularly disposed of outside institutional frameworks with many HHC patients placing potentially contaminated sharps in domestic waste [24]. The general approaches to waste disposal for Community Healthcare Practitioner-Led and Patient-Led sharps management are compared in Figure 2.

The disposal procedure for sharps waste within clinical settings and by community nurses follows a well-regulated pathway whereby the filled containers are removed, at least in principle, by experienced waste contractors. Needle use and disposal by self-injecting patients is, however, more complex and is subject to a myriad of human attitudes and perceptions in combination with logistical factors. There are obvious detractors such as the need to procure the sharps container and to return it to a designated collection facility. In addition, there will be inevitable issues over costs associated with the supply and transport of the container. It is also likely that a proportion of patients may simply dismiss the seriousness of the sharps hazard.

A worrying trend that has arisen among the diabetic community, however, is for the reuse of insulin needles and lancets despite warnings over the possibility of infection and the fact that each use leads to the mechanical deformation of the needle tip, which increases tissue scarring (lipodystrophy) and can increase the risk of metallic fracture and fragments being left in the skin [25]. A survey by Costello and colleagues found that when interviewing diabetic patients on their approach to sharps waste, 86% did not follow the proper procedure with the majority simply placing discarded needles within the household trash [26]. Furthermore, 7% disposed of their waste needles by means of the toilet. The fact that the needles are placed within normal plastic bags ill-equipped to deal with potential needle puncture or are flushed to waste presents clear hazards for the workers collecting domestic waste or dealing with sewer drainage or downstream water treatment [26]. 

## 3. Engineering a Solution to Needlestick Injuries

The provision of safety engineered devices (SEDs) to improve sharps safety has been widely advocated as a means through which to safeguard against potential injury [5] and there is no doubt that the introduction of needle enclosures that disarm the sharp (through retracting, sheathing, or blunting) combined with the increased accessibility of sharps containers have played a significant part in reducing the incidence of injury within the workplace [1,2]. Yet, it would appear, at least on the basis of the CDC figures, that disposal accounts for some 60% of NSI events [4]. More worrying is the fact that a significant proportion (5%) of NSIs arise through enacting the disarming mechanism that is intended to render the sharp safe. The results of the Massachusetts NSI surveillance study (2007) found that, of those subject to injury, 65% did not use any needle safety device and, of the 31% that did, 28% cited device failure among the causative factors [3]. A survey of community nurses by Quinn and coworkers (2009) found a variety of negative attitudes towards the safety devices where they were perceived to be too difficult to use (26%), did not work well (24%), and were generally too time consuming to apply (7%) [16]. It is clear from many similar reports that there is still a considerable need for the design of more effective and accessible safety solutions.

## 4. The Quest for Needle-Free Delivery

**Figure 3:**
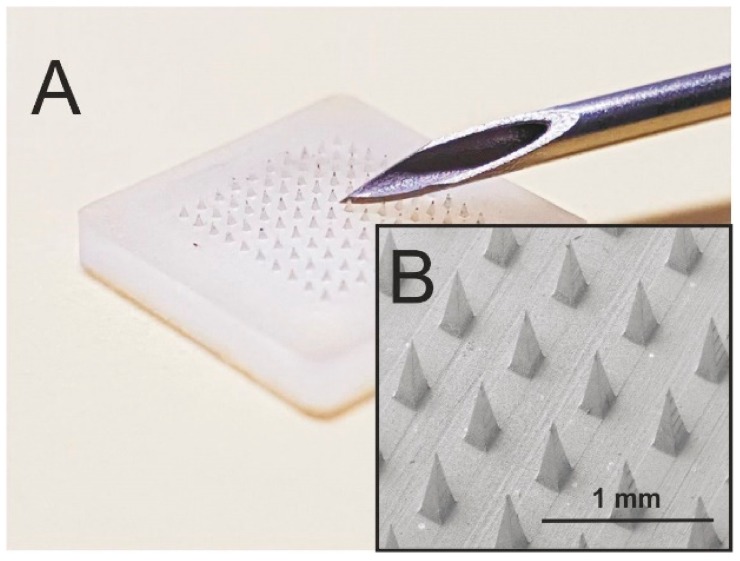
(**A**) Comparison of a microneedle patch (200 × 200 × 350 μm) with a standard hollow-bore needle. (**B**) Electron micrograph of the polystyrene microneedle patch.

The introduction of needle-free transdermal delivery systems has long been an aspiration but it has become more of a reality in recent years. Microneedle technologies in particular have risen to the fore and have captured the attention of the healthcare community as they profess to eliminate the issues of needle phobia but, more importantly, counter a raft of safety issues normally associated with needle disposal. Microneedle systems are designed to puncture the outer skin layers and allow the passage of a drug directly through to the underlying microcirculation [27]. These tend to have an array of micron-sized projections (50–900 μm long), as highlighted in Figure 3. The needles typically need to be of a minimum length in order to overcome the elastic deformation of the epidermis. This was demonstrated by Verbaan and co-workers (2007), where movement of the skin during the application/insertion of the microneedle patch composed of 300 micron needles failed to puncture the skin barrier [28]. In general, the actual penetration depth is markedly less than the overall length of the actual microneedle.

### 4.1. Microneedle Designs and Implications

**Figure 4:**
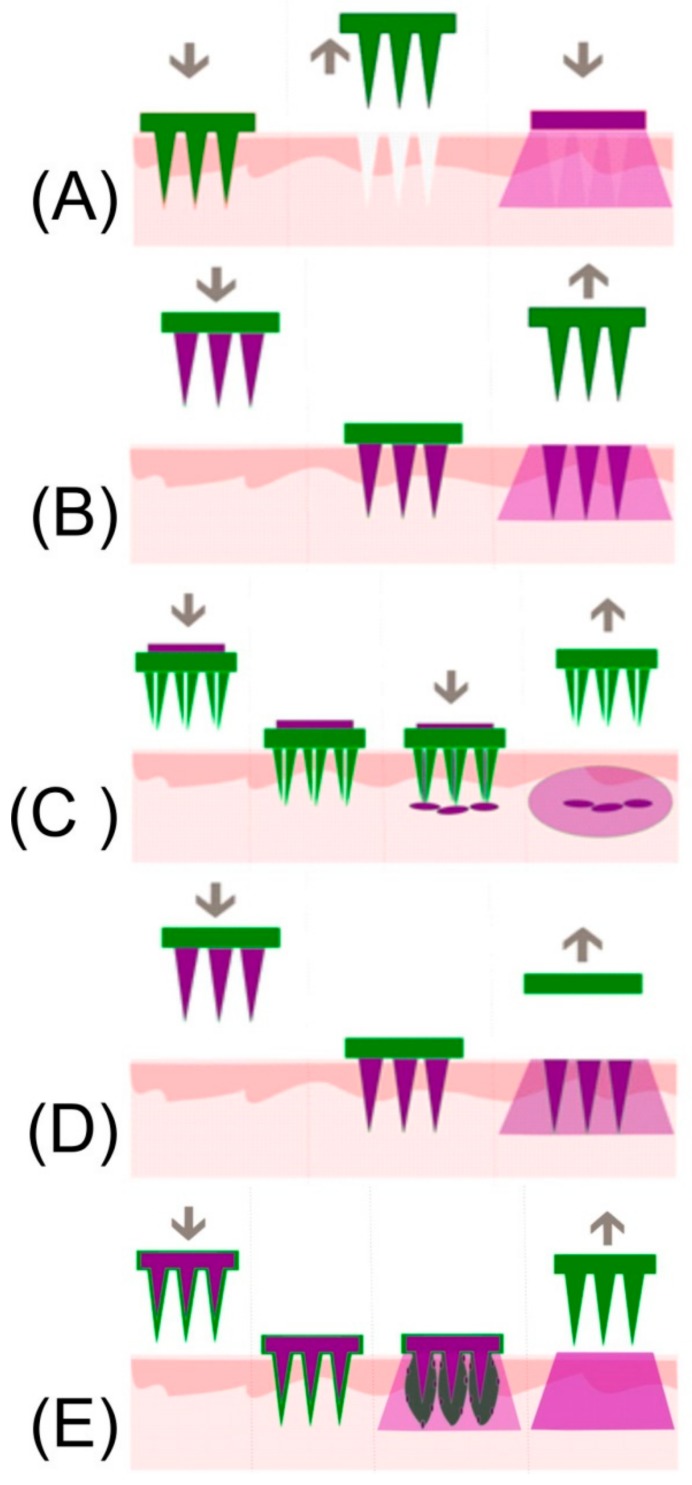
Mode of action inherent to solid (**A**), coated (**B**), hollow (**C**), dissolvable (**D**), and swellable (**E**) microneedle systems. (Reproduced with permission from [29]).

The central rationale behind the development of microneedles as a delivery system arose from the desire to offer pain-free administration, associated with transdermal routes, whilst creating microchannels that breach the hydrophobic skin barrier and thereby enable a far greater range of drugs to be transported [29,30]. Recent investigations of user perceptions and direct experience of microneedle patches found that the majority of those surveyed described their application as a ‘pressing,’ or ‘heavy’ sensation in comparison to the ‘sharp’ and ‘stabbing’ feeling arising from conventional hypodermic injection [31]. Studies comparing the skin sensation of applying a flat baseplate with that of a microneedle patch found that only 20% of the volunteers were able to distinguish between the two and confirmed that the needles are generally sufficiently short to avoid activation of the underlying dermal nerve network [32]. There are five basic approaches commonly used in the design of microneedle drug delivery systems and are based on: solid, coated, hollow, dissolvable, and swellable designs. The mode of action for each design rationale is summarised in Figure 4 and the features and safety issues associated with each are described briefly within the following sections.

Irrespective of the administration route or the mode of operation, the physical damage to the skin is minimal as a consequence of the small dimensions of the needles and, under non-occlusive conditions, the channels close within 2 h of the original treatment [33]. It has been shown, however, that the lifetime of the micropore can be extended through chemical manipulation where the introduction of diclofenac and fluvastatin as co-eluting drugs can delay closure by up to seven days [34,35]. It has been postulated that the resulting microchannels, and any delay in closure, could effectively serve as a highway to the underlying tissue and microcirculation for bacteria. It can be anticipated that the implementation of good clinical practice (i.e., swabbing the area with antibacterial wipes such as alcoholic chlorhexidine, etc.) prior to exposure can often preclude the influx of adventitious species that may be present on the surrounding skin. A number of studies, however, have demonstrated that the potential for infection arising from microneedle application is considerably less when compared with conventional injection systems [36,37]. Advances in polymer processing have also seen the development of needles that possess an intrinsic antibacterial action which can further reduce the threat of infection from microbes that may have been inadvertently drawn into the channel during application of the patch [38]. Despite such innovations, the potential transmission of BBPs as a consequence of accidental puncture still remains.

### 4.2. Solid Microneedles/Coated Microneedles

Solid microneedles based on silicon, titanium, or stainless steel were among the first to be investigated for use within drug delivery systems whereby the needles are applied to the skin through a discrete patch, or by mean of a punch or roller that are commonly referred to as “poke and patch” approaches [39]. Such systems are similar to the dermabrasion rollers used in cosmetic treatments which have received interest from regulators concerned with the transmission of BBPs. More recent designs have focused on coating the needle arrays with a therapeutic agent [40]. Once the patch is applied, the needles breach the SC layer, allowing the drug molecules to simply dissolve into the surrounding tissue and diffuse to the microcirculation. The dosage transferred ultimately depends on the microneedle area onto which the drug can be coated and therefore the potential yield will be limited. The use of coated microneedles has found a particular niche in the delivery of agents with a low yield-high potency profile (i.e., antigenic material/RNA) [41]. The exploitation of microneedles as a vaccine delivery system is particularly pertinent as there is a significant population of antigen-presenting cells (APCs) within the outer skin layers which enables a strong immune response to be obtained from the delivery of small amounts of immunogenic material [42]. Ahmad and coworkers (2015) critically reviewed the various processes through which microneedles can be coated to optimise the delivery yield [43]

It has been shown that the microneedle approach in such contexts results in comparable or, in some cases, superior performance to conventional subcutaneous and intramuscular injections [44,45]. Crucially, the approach deftly avoids many of the safety concerns associated with conventional hypodermic needles and avoids issues of patient needle phobia. The latter can be especially important when considering the vaccination of young children and there have been numerous studies comparing the preference of patch to conventional needle systems. An interesting note is that it has been postulated that self-administered patches could improve vaccination uptake. This assertion has been supported in a recent study where the availability of the latter was found to increase intent to be vaccinated from 44% to 65% when compared with the standard injection modes [46,47].

### 4.3. Hollow Microneedles

Hollow microneedles have been developed to allow the transfer of a drug from a reservoir within the patch to the microcirculation and can enable volumes of up to 200 μL to be transported. In many respects, the system mimics the operation of a conventional hypodermic syringe [42,48,49,50,51]. It must be noted that hollow needle fabrication is significantly more complex and those possessing a high aspect ratio lack the internal support structure common to solid needles, leading to potential failure if improperly inserted. It can be expected that random movements during manipulation of the patch assembly or device, during both insertion and removal, will result in a variety of stresses (axial compression and sheer) which may lead to the failure and fracture of the needles [52,53,54,55,56]. The latter can be significantly complicated by natural variations in morphology of the patient’s skin, where non-uniform insertion of the microneedle array will inadvertently induce sheer stress and cause transverse bending of the microneedle structures [57,58]. Most approaches to the manufacture of microneedles acknowledge that a decrease in the microneedle height counters many structural stress issues and provides a more favourable safety margin [57].

### 4.4. Dissolvable and Swellable Microneedles

One of the more recent strategies to emerge in microneedle design is based on the use of dissolvable polymers. The rationale here that the drug to be delivered is entrapped within the core of the needle at the time of fabrication. Upon breaching the skin, the polymer that forms the architecture of the needle dissolves and thereby releases a drug. The dissolution of the needle within the skin effectively eliminates the possibility of post-application NSI [59,60,61,62,63,64,65,66]. As with coated solid microneedles, a core restriction relates to the delivery yield as the primary drug delivery component is the needle structure and, unlike the hollow needle designs, the base plate does not normally act as a reservoir [27]. This introduces a degree of complexity in the manufacture as there must be a balance between the amount of drug to be delivered and the amount of polymer required to ensure the structural integrity of the actual needle [58]. Liu and co-workers (2014) found that in the case of insulin, the maximum drug content was around 10%, giving a delivery yield of 0.6 mg per cm^2^ [66]. 

Microneedles that swell after puncturing the stratum corneum are a further refinement and aim to address the low yield restrictions of the dissolvable designs. The patches are based on a hydrogel structure whose hydrophilicity actively absorbs fluid from the surrounding tissue, resulting in an expansion of the needle core creating pores and nano-channels through which the therapeutic agent can diffuse [41]. A core advantage of this approach centres on the use of the baseplate as a reservoir for the drug which is capable of transport through the swollen microneedle structure to the underlying microcirculation. Some of the more recent developments in dissolvable and swellable microneedle systems are highlighted in Table 1 and it can be seen that there is a wide variety of drug candidates and potential applications. A particularly innovative translation of the swellable system is the ability to extract interstitial fluid [67,68,69]. Given that most blood sampling involves IV extraction, the swellable MN system offers painless removal of fluid whilst the swollen needles remove the possibility of post-sample needlestick.

The time taken from skin puncture to channel closure has been shown to be of the order of several hours for conventional, solid needles but the situation can become more complex with the dissolvable/swellable systems in situations where undissolved polymer residues may be left behind and continue to transcend the skin barrier. There can be little doubt that rapid healing of the puncture microchannels will be a major factor in minimising infection but this could be compromised where there is a failure in the dissolution or removal of a swellable microneedle. There is scant information available on the mechanical failure of such systems and the consequences for restoring skin integrity but this reflects the fact that research within this particular niche is only just emerging. The emphasis is still on the technological advances inherent in the design and material functionality and it is inevitable that implications for skin function will begin to emerge in the future.

## 5. Needle Handling and Disposal

The disposal of sharps has been a perennial safety issue even within standard, well-regulated environments [83]. On a local level, sharps collection containers are undoubtedly the most visible example of safety control measure; however, their effectiveness in minimising injury depends on the responsibility of the user to comply with good practice [15]. Sadly, their misuse has led to the recognition of “container-associated sharps injury” (CASI) as a new hazard category and, presently, it is estimated that it alone accounts for 5–6% of annual sharps injuries [84]. Most recorded incidents are attributed to being struck by the sharp being disposed at the time (53%) or from sharps that protrude from the container opening for reasons other than over-filling (33%) and, in most cases, the causative factor is largely carelessness in the disposal process [8,26,84]. On a local level, where there is a need for repeated injection (i.e., insulin), the frequency and associated bulk means that there will be a corresponding increase in the frequency with which sharps containers must be replaced and, particularly from a Home Healthcare perspective, can be a reason for over-filling and hence accidental NSI. Difficulties with procuring a container, facilitating its removal, and the associated costs are significant factors in the failure to observe appropriate safety measures and lead to disposal through domestic trash.

Blenkharn and Odd (2008) conducted a detailed analysis of a specialist waste contractor and found that most issues (85%) arise where there is inadequate segregation of soft wastes (dressings, etc.) from sharps [85]. They found that the latter invariably end up in thin plastic refuse sacks that afford no puncture protection to the housekeeping staff and waste handlers, with incomplete closure of the sharps containers accounting for the remainder (15%). The main cause of injury was attributed to hypodermic syringes (92.5%), with puncture wounds being the most common. The overall incident rate was 1 per 29,000 man hours but this was for an organisation with employees well versed in the hazards associated with medical waste and with access to appropriate personal protection equipment (PPE). A study by Turnberg (1996) reported that in the US, workers handling clinical waste had a 2.4–7 times greater chance of getting infected by HIV compared to other staff in healthcare facilities [86]. The hazard is compounded by the cost associated with the treatment and disposal of medical waste, whereby the latter can be prone to purposeful misclassification and improper disposal [87,88,89]. It has been estimated that general waste costs some 10–20 times less to dispose of than clinical waste [89].

It must be noted from the investigation by Blenkharn and Odd that a prime contributor to the subsequent injury was the failure of the employee to apply the correct PPE [85]. When considering domestic trash originating from Home Healthcare, it is likely that the movement from a house waste bin to an external collection bin will be conducted by a family member or helper who will be unprepared should there be the possibility of a NSI. Skin puncture from a protruding needle will clearly raise the possibility of BBPs but the significance of the injury may be lost and, as such, there may well be no serological testing nor post-exposure prophylaxis. Given that speed is crucial in the application of the latter, the consequences of a delay in follow-up could be life threatening. Likewise, there will be latent risk for those municipal workers handling the collection of the waste [90,91]. Microneedle patches, particularly those based on dissolvable/swellable designs would clearly avoid many of the issues associated with conventional needle disposal and as the sharp component is largely removed at the time of application—this would effectively eliminate the threat of subsequent NSI and the transmission of a BBP.

It is also important to rationalise the impact of the microneedle system on the volume of waste. In principle, it would remove the need for specialised sharps containers (thereby removing CASI) and, in principle, would enable their disposal in general waste. The latter has increasing significance where the volume of waste associated with conventional sharps and their segregation into specialised categories creates a considerable economic burden for healthcare administrators. An example of the magnitude was given by Emmanuel and colleagues (2004), who reported that a relatively small vaccination campaign in the Philippines gave rise to over 130 tonnes of sharps waste [92]. It must also be noted that in less developed countries, the disposal of medical waste onto municipal sites will also dramatically increase the risk of NSI where scavenging occurs and the recycling of the medical wastes is not uncommon [90,93]. The use of microneedle patches to remove the NSI threat in such contexts cannot be overstated.

It is hard to envisage any threat from the microneedles, but that perception can be hazardous in itself. There is an increasing interest in the use of microneedle systems for diagnostics [94] which will inevitably be based on solid, non-dissolvable systems. As such, they can still constitute a sharps hazard much in the same way that dermarollers have given rise to caution [12]. The removal of a patch in which the needle array is still intact could present a hazard for BBP transmission. Moreover, as there is little or no sensation upon microneedle puncture, there may be no recognition that an NSI has taken place and, as such, there will be no follow-up investigation, treatment, or counselling. It must be appreciated, however, that the actual risk of BBPs from a microneedle patch will be very limited. The transmission of a BBP from a hollow-bore syringe needle has been estimated to be some 10 times greater than that through contact with a solid needle (i.e., a suture needle). The disparity here relates primarily to the smaller volume held by the solid surface [95,96]. Clearly, the area of the microneedle in contact with the tissue will be very small and thus the liquid-holding capacity (at least in terms of solid microneedle) will be very small.

## 6. Conclusions

Microneedle patches have evolved as an alternative to conventional hypodermic injections in a number of clinical applications largely on the basis that they can offer pain-free drug delivery that counters the apprehension that conventional hollow bore needles generate. The latter is particularly pertinent when considering paediatric vaccinations where the more “Velcro”-like perception of the needle patch can help dispel anxiety and actually enhance participation. It is clear that microneedles possess enormous promise and could radically reduce the potential for needlestick injury. There remain many challenges in microneedle design—especially in relation to drug yield and, while the devices cannot completely replace conventional hollow-bore needles, they have the capability of making significant in-roads in drug delivery practice.

## Figures and Tables

**Table 1:** Recent developments in dissolvable/swellable microneedle systems.

Drug	Polymer	Type	Ref.
Dihydroergotamine mesylate	Polyvinylpyrrolidone	D	[70]
Thymopentin	Polyvinylpyrrolidone	D	[71]
Exendin-4	Carboxymethylcellulose	D	[72]
Fluorescent Model	Hyaluronic acid/PVA	D	[73]
Sumatriptan succinate	Polyvinylpyrrolidone	D	[74]
Adenosine	Hyaluronic acid	D	[75]
Vitamin K	Gantrez^®^ S-97, a copolymer of methyl vinyl ether and maleic acid	D	[76]
Lysozyme	Polyvinylpyrrolidone	D	[77]
Valproic acid	Carboxymethylcellulose	D	[78]
Besifloxacin	Polyvinylpyrrolidone	D	[79]
Caffeine/Theophylline	Hydrolysed poly(methyl-vinyl ether-co-maleic anhydride) and poly(ethyleneglycol)	S-E	[67]
None Specified	Poly(methyl vinyl ether-co-maleic acid) and pectin	S	[80]
Glucose/Cholesterol	Methacrylated hyaluronic acid	S-E	[68]
FITC-dextrans	Silk fibroin	S	[81]
Curcumin	Gantrez^®^ S-97 poly(methyl vinyl ether-co-maleic acid) and Tween 85	S	[82]

Where: D = dissolving; S = swellable; S-E = swellable extraction of fluid.
